# Bosutinib Inhibits EGFR Activation in Head and Neck Cancer

**DOI:** 10.3390/ijms19071824

**Published:** 2018-06-21

**Authors:** Carmen Segrelles, David Contreras, Elena M. Navarro, Carmen Gutiérrez-Muñoz, Ramón García-Escudero, Jesús M. Paramio, Corina Lorz

**Affiliations:** 1 Molecular Oncology Unit, CIEMAT (ed 70A), Ave Complutense 40, 28040 Madrid, Spain; contreras.david.1987@gmail.com (D.C.); elena.np92@gmail.com (E.M.N.); carmen.gutierrezm@quironsalud.es (C.G.-M.); ramon.garcia@ciemat.es (R.G.-E.); jesusm.paramio@ciemat.es (J.M.P.); 2 Molecular Oncology, University Hospital 12 de Octubre, Research Institute 12 de Octubre i+12, Ave Córdoba s/n, 28041 Madrid, Spain; 3 Centro de Investigación Biomédica en Red de Cáncer (CIBERONC), 28029 Madrid, Spain

**Keywords:** head and neck cancer, targeted therapies, EGFR inhibitors, Bosutinib, Alpelisib, cancer cell lines

## Abstract

Head and neck squamous cell carcinoma (HNSCC) is the sixth most common cancer worldwide, and although new therapeutic approaches have been recently evaluated, overall patient survival is still poor. Thus, new effective and selective clinical treatments are urgently needed. An analysis of data from large-scale, high-throughput drug screening cell line projects identified Bosutinib, a Src/Abl inhibitor that is currently used for the treatment of chronic myelogenous leukemia, as a candidate drug to treat HNSCC. Using a panel of HNSCC-derived cell lines, we found that treatment with Bosutinib reduced cell proliferation and induced apoptosis of sensitive cell lines. The drug rapidly inhibited Src and EGFR (epidermal growth factor receptor) phosphorylation, and sensitivity to Bosutinib was correlated with the activation status of EGFR. Similar findings were observed in in vivo xenograft assays using HNSCC derived cells. Moreover, in the presence of mutations in *PIK3CA*, the combination of Bosutinib with the PI3Kα inhibitor Alpelisib showed a synergistic effect. These results suggest that Bosutinib could be a new effective drug for the treatment of HNSCC, particularly in tumors with high EGFR activity. Its combination with Alpelisib could especially benefit patients bearing activating mutations of *PIK3CA*.

## 1. Introduction

Head and neck cancer arises in the oral and nasal cavities, pharynx and larynx, and in more than 90% of cases, is of squamous origin. Head and neck squamous cell carcinoma (HNSCC) is the sixth most frequent cancer in the world with an incidence rate of ~600,000 cases per year [1]. The long-term survival rates are alarmingly low, with half of the patients dying within 5 years [2] and 10–40% of patients who experience recurrent or metastatic disease dying within a year [3]. The therapeutic options for HNSCC patients are limited. Novel approaches targeting potentially critical pathways are needed to overcome these low survival rates.

Due to the increasing understanding of the molecular mechanisms and basic pathways in the pathogenesis of HNSCC, current research is focused on molecularly-targeted therapies specific for the pathways involved in the carcinogenesis of HNSCC, such as the epidermal growth factor receptor (EGFR) and phosphatidylinositol 3-kinase (PI3K) pathways. The tyrosine kinase epidermal growth factor receptor (EGFR) is a well-characterized oncogene in HNSCC. It plays important roles in normal cell growth, lineage determination, repair and functional differentiation. *EGFR* is frequently altered by activating mutation, amplification and/or overexpression in ~25% of the tumors [4]. It correlates with poor responses to treatment, increased tumor growth, metastasis and resistance to chemotherapy and radiation therapy [5]. In fact, Cetuximab, a monoclonal, anti-EGFR antibody that binds to EGFR and prevents activation of the downstream signaling pathway, was, until recently, the only approved targeted agent for HNSCC therapy. This medicine can inhibit cell growth and survival and has demonstrated overall survival improvements in clinical trials when combined with radiotherapy or chemotherapy [6,7]. However, the overall increased response to this drug has been lower than initially expected, in part because some patients develop resistance to Cetuximab after an initial benefit. Several studies have identified refractory mechanisms that bypass the inhibition of the EGFR pathway, providing an explanation for the resistance to therapy [8]. Because of this, new drugs targeting the pathway in a different way as well as co-targeting strategies are under investigation. Another cell-growth pathway altered in HNSCC is the PI3K/Akt/mTOR, with *PIK3CA* (phosphatidylinositol-4,5-bisphosphate 3-kinase catalytic subunit α) being the most commonly altered gene. This pathway regulates similar processes to those described for EGFR. *PIK3CA* encodes the catalytic subunit α of class IA PI3K (PI3Kα, phosphatidylinositol 3-kinase α) and is affected in ~55% of cases. Activating mutations in *PIK3CA* have been found in ~20% of HNSCC cases with hot-spot E543K, E545K and H1047R substitutions being the most common [4,9,10].

Based on the analysis of large-scale drug sensitivity screening studies [11], Bosutinib was identified as a candidate drug for HNSCC treatment [12,13]. Bosutinib is an orally-active, ATP-binding site competitive inhibitor of Src and Abl kinases. It was approved for the the treatment of Philadelphia chromosome positive chronic myelogenous leukemia by the Food and Drug Administration (FDA) in 2012 [14]. It shares a similar structure to Gefitinib and Erlotinib, which are both FDA-approved EGFR specific tyrosine kinase inhibitors that are under clinical trials for HNSCC [15] (Available online: http://clinicaltrials.gov). A recent study of Src inhibitors confirmed the capability of Bosutinib to inhibit kinases beyond the Src family, directly inhibiting EGFR [16].

In this study, we found that sensitivity to Bosutinib in HNSCC cell lines is dependent on increased EGFR activity. Additionally, we showed that Bosutinib inhibits EGFR activation in vivo in a HNSCC xenograft model. The combination of Bosutinib with the PI3Kα inhibitor Alpelisib, which has shown good efficacy and tolerability in several cancers, including HNSCC [17,18,19], efficiently inhibited both EGFR/ERK and PI3K pathways in HNSCC cell lines. Our results support Bosutinib as a therapy in HNSCC patients, either alone or in combination with Alpelisib in the context of *PIK3CA* mutations.

## 2. Results

### 2.1. Sensitivity of HNSCC Cell Lines to Bosutinib

**Figure 1:**
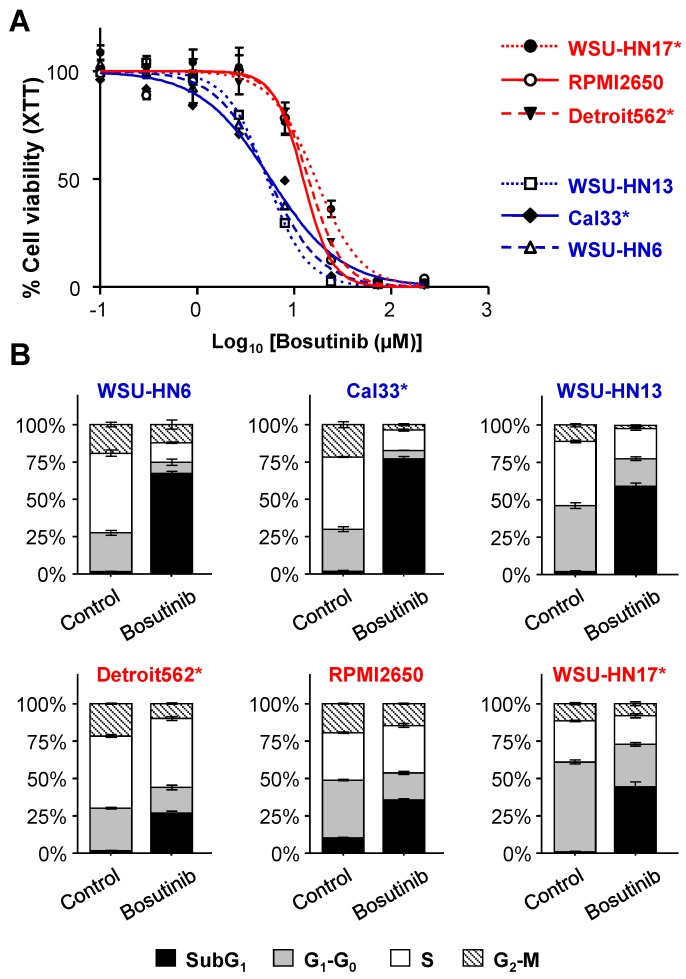
Sensitivity of head and neck squamous cell carcinoma (HNSCC) cell lines to Bosutinib. (**A**) Cell viability as measured by XTT. Data represent means ± SEMs from a representative experiment of at least three different experiments for each cell line (each concentration point was replicated six times within each experiment). The cell viability curves (continuous, broken or dotted) for each cell line are a non-linear regression fit of the data (log inhibitor versus normalized response). (**B**) Cell cycle analysis of Bosutinib-treated HNSCC cell lines. Cells were treated with their corresponding IC_75_ for 24 h and the cell cycle was analyzed by flow cytometry. The graphs represent the percentage of cells in each phase of the cell cycle. Data represent means ± SEMs from at least two different experiments (each point was replicated three times within the experiment). * Cell lines with mutations in *PIK3CA*. In red are cell lines resistant to Bosutinib; in blue are cell lines sensitive to Bosutinib.

We analyzed the sensitivity to Bosutinib in a panel of HNSCC-derived cell lines (Table 1). To cover some of the breadth and complexity of this tumor type, we chose well-characterized cell lines from different head and neck origins, including locoregional (lymph node) metastasis as well as oncogenic alterations commonly found in this type of cancer, such as *EGFR* overexpression or *PIK3CA* activating mutation. Our results showed that Bosutinib decreases cell proliferation (Figure 1A) and induces apoptosis in HNSCC cell lines (Figure 1B), which is in agreement with other tumor-derived cell lines [13,16,20,21]. The IC_50_ of three of the six cell lines studied—WSU-HN6, Cal33 and WSU-HN3—was nearer to the range of peak plasma concentration reached in patients treated with doses of the drug used for cancer therapy [22] (Figure 1A, Table 2); thus, we defined these three cell lines as sensitive, while Detroit562, RPMI2650 and WSU-HN17 were defined as resistant. In Bosutinib-sensitive cell lines, the dose of Bosutinib causing a 75% decrease in cell viability (IC_75_ as measured by XTT) caused a similar amount of apoptotic cell death as measured by the percentage of cells with SubG_1_ content in the flow cytometry analysis of the cell cycle (Figure 1B and Table 2). This was not the case for the resistant cells, in which the percentage of apoptotic cells was lower, and the decrease in cell viability could be, at least in part, due to an arrest in the progression of the cell cycle.

### 2.2. Sensitivity to Bosutinib Correlates with Phosphorylated EGFR Protein Levels

**Figure 2:**
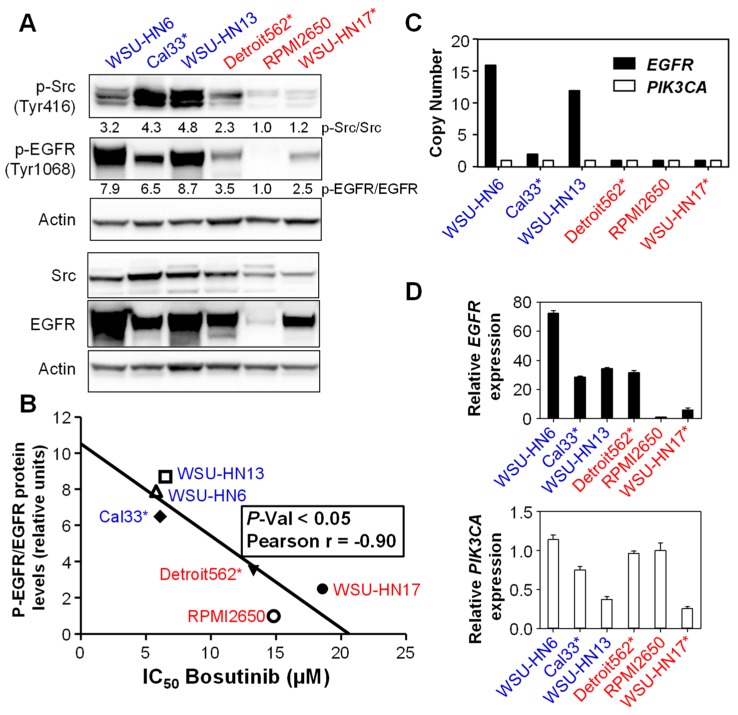
(**A**) Src and epidermal growth factor receptor (EGFR) phosphorylation was analyzed by Western blot analysis. The numbers indicate the normalized ratio of the phosphorylated form versus the total form. (**B**) Linear regression of the p-EGFR/EGFR ratio versus sensitivity to Bosutinib (IC_50_) of the different HNSCC cell lines. *EGFR* and *PIK3CA* copy number (**C**) and mRNA expression (**D**). * Cell lines with mutations in *PIK3CA*. In red are cell lines resistant to Bosutinib; in blue are cell lines sensitive to Bosutinib.

Next, we sought to identify a possible biomarker of sensitivity to Bosutinib in the HNSCC cell lines. We studied the protein and phosphoprotein levels of Src and EGFR in these cells (Figure 2A), and we observed that the relative EGFR activity level (p-EGFR/EGFR) correlated better with sensitivity to the drug (Figure 2B) than that of Src. This is consistent with the observation that Src inhibitors, and in particular Bosutinib, are able to inhibit EGFR activation at levels comparable to the specific EGFR inhibitor Erlotinib [16]. No mutations in the *EGFR* gene have been reported in the cell lines used in this study. Thus, we studied the copy number variation (CNV) and expression of the *EGFR* gene (Figure 2C and D upper panel). WSU-HN6, Cal33 and WSU-HN3 Bosutinib-sensitive cell lines showed increased *EGFR* gene copy number and expression, which are both frequent events in HNSCC [4], as compared to Bosutinib-resistant cell lines. Among the resistant cell lines, the Detroit562 and WSU-HN17 cells showed similar levels of total EGFR protein to Cal33; however, neither of them had amplification of *EGFR*, and only Detroit562 showed a comparable level of *EGFR* gene expression. Importantly, EGFR phosphorylation levels were lower in Detroit562 and WSU-HN17 cells compared to Cal33.

*PIK3CA* alterations, such as mutations and gene amplification and overexpression are common in HNSCC [4,9,10,23,24,25]. Three of the six cell lines studied had activating mutations in this oncogene (Table 1), but the mutations did not seem to yield a differential effect on their sensitivity to Bosutinib (Figure 1). None of the cell lines displayed CNV in *PIK3CA* (Figure 2C). Also, *PIK3CA* gene expression was similar among them (Figure 2D, lower panel).

### 2.3. Bosutinib Effectively Inhibits Src and EGFR Phosphorylation in HNSCC Sensitive Cell Lines and in Xenografts

**Figure 3:**
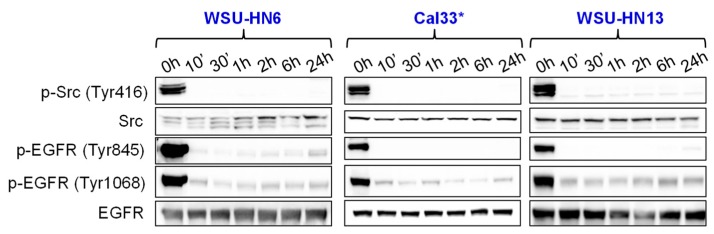
Western blot analysis of Src and EGFR phosphorylation of Bosutinib-sensitive HNSCC cell lines treated with the IC_50_ of the drug at different time points. * Cell lines with mutations in *PIK3CA*.

To better understand the effect of Bosutinib on HNSCC cell lines, we studied Src and EGFR tyrosine kinase in cells treated with the IC_50_ dose of the drug at different times. In Bosutinib-sensitive cells, Src activating phosphorylation at Tyr416 was inhibited at early time points (10 min) and the inhibition was maintained at later time points (24 h) (Figure 3). Similarly, EGFR phosphorylation at Tyr845 and Tyr1068 was inhibited at early and late time points. It has been well described that Src phosphorylates EGFR on Tyr845 [26]; thus, Bosutinib inhibition of Src activation could mediate this decrease in p-EGFR Tyr845. However, phosphorylation of EGFR at Tyr1048 occurs upon ligand-activated autophosphorylation, and EGFR autokinase activity is not dependent on the Tyr845 phosphorylation status [27]. This suggests that Bosutinib could exert a direct effect on EGFR in HNSCC sensitive cell lines.

**Figure 4:**
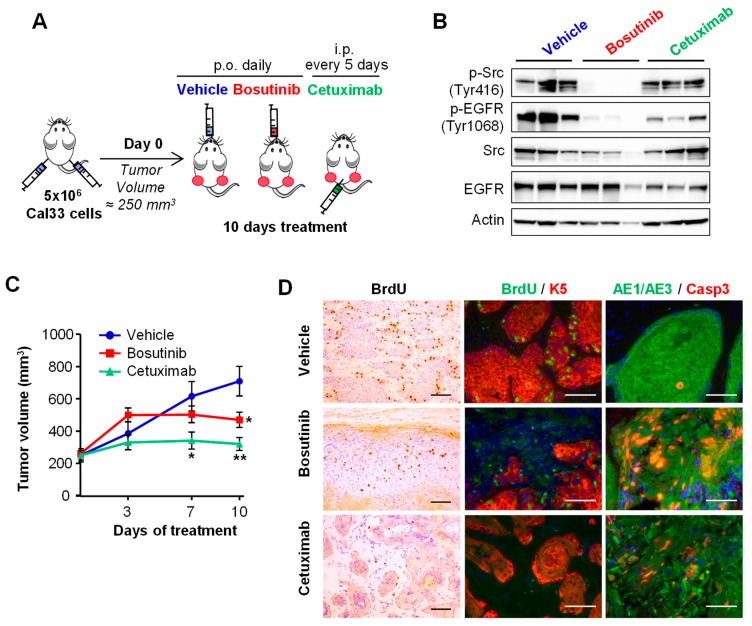
Effect of Bosutunib in HNSCC xenografts. (**A**) Cal33-derived xenografts were treated as indicated. P.o.: *Per Os* (oral administration); i.p.: intraperitoneal. (**B**) Western blot with the indicated antibodies of lysates from tumors treated as specified for 10 days. Each lane is a lysate from a tumor corresponding to the indicated treatment group. (**C**) Tumor growth of Cal33-derived xenografts treated as indicated (*n* = 10 per arm). * *p* < 0.05, ** *p* < 0.01. (**D**) Sections from tumors treated as indicated for 10 days were immunostained with BrdU to assess proliferation, the epithelial marker keratin 5 (K5), the pan keratin marker AE1/AE, and the apoptotic marker Caspase 3. Scale bar 100 µm.

To further characterize the effects of Bosutinib in the EGFR blockade, we conducted studies in HNSCC xenografts. Animals bearing Cal33-derived xenografts were treated with Bosutinib or with the EGFR monoclonal antibody drug Cetuximab, as indicated (Figure 4A). Bosutinib inhibited Src and EGFR phosphorylation in the tumors; moreover, Bosutinib-mediated EGFR phosphorylation inhibition was comparable to the effects of Cetuximab (Figure 4B). Both Bosutinib and Cetuximab inhibited tumor growth after 10 days of treatment (Figure 4C). An immunohistochemical analysis of the tumors at this time point revealed that both treatments decreased tumor proliferation and increased cell death (Figure 4D).

### 2.4. Bosutinib and PI3Kα Inhibitors Show a Synergistic Effect on Cell Viability in the Presence of Mutations in PIK3CA

**Figure 5:**
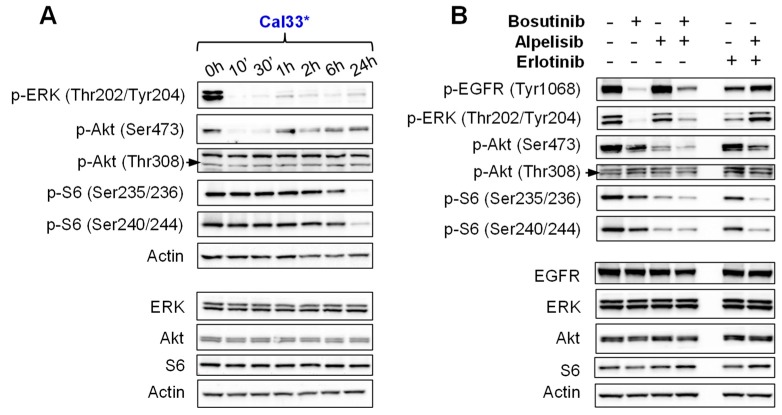
(**A**) Western blot analysis of ERK, Akt and S6 phosphorylation of Cal33 cells treated with the IC_50_ of Bosutinib at different time points. (**B**) Western blot analysis of ERK, Akt and S6 phosphorylation of Cal33 cells treated with Bosutinib (IC_50_), Alpelisib (IC_50_) and Erlotinib (10 µM) at 6 h. * Mutated in *PIK3CA*. Black arrows indicate p-Akt Thr308.

Subsequently, we studied the effect of Bosutinib on EGFR downstream signal transducers in sensitive cells. Bosutinib was more effective at inhibiting the phosphorylation of ERK than the phosphorylation of Akt. Phosphorylation of the mTOR downstream target S6 decreased only at later time points (Figure 5A, Supplementary Figure S1A). The combination of Bosutinib with PI3Kα inhibitors (Alpelisib or Dactolisib) efficiently inhibited the phosphorylation of ERK, Akt at Ser473, and S6 (Figure 5B, Supplementary Figure S1B,C). In fact, Bosutinib and the combination of Bosutinib and Alpelisib were more effective at inhibiting the downstream signaling of EGFR than Erlotinib alone or together with Alpelisib (Figure 5B, Supplementary Figure S1B,C).

**Figure 6:**
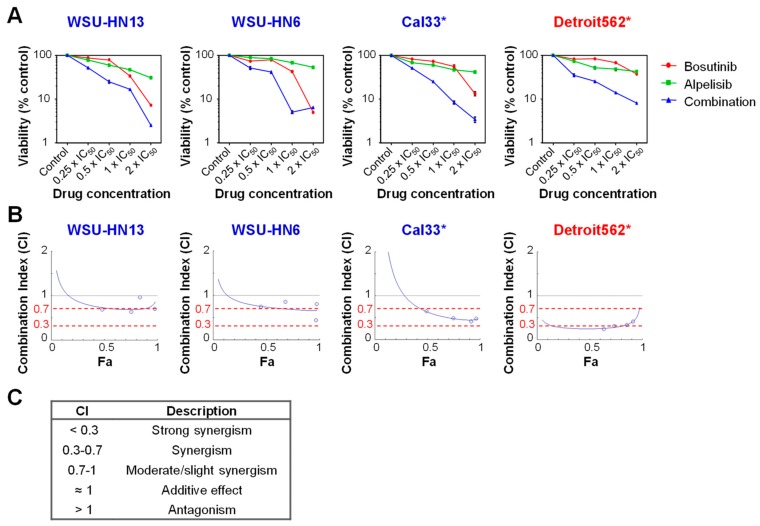
Sensitivity of HNSCC cell lines to Bosutinib in combination with Alpelisib. (**A**) Cell viability, as measured by XTT of the indicated cell lines treated with Bosutinib, Alpelisib and their combination at the indicated amounts of their IC_50_ dose. Data represents means ± SEMs (each concentration point was replicated four times within the experiment). (**B**) Fa–CI plots to measure the synergism of the combination of two drugs. Fa represents the fraction of the cells affected by the combination of both drugs (at the concentrations indicated in panel A), “0” being 100% cell survival and “1” being 0% cell survival. The Combination Index (CI) was calculated using Compusyn software and plotted for each combination. (**C**) Description of the CI values. * Cell lines with mutations in *PIK3CA*. In red are cell lines resistant to Bosutinib; in blue are cell lines sensitive to Bosutinib.

The combination of targeted therapies to block multiple molecular pathways is an effective strategy to prevent or delay acquired resistance. In this context, we studied the effects of inhibiting both EGFR and PI3Kα on HNSCC cell lines. For this purpose, we chose two cell lines wild type for *PIK3CA* (WSU-HN13 and WSU-HN6) and two with mutation in this gene (Cal33 and Detroit562). The combination of Bosutinib and Alpelisib caused a greater reduction in cell viability than the effect of either drug alone (Figure 6). However, simultaneous inhibition of EFGR and PI3Kα resulted in an additive effect or moderate synergism in *PIK3CA* wild type cells and had a clear synergistic effect in *PIK3CA* mutant cell lines, irrespective of their sensitivity to Bosutinib (Figure 6B,C, Table 2).

## 3. Discussion

The World Health Organization predicted that there will be 833,424 new HNSCC cases in 2020, about 100,000 more cases than in 2015. Despite the latest advances in the therapy for this cancer, the mortality rate remains unchanged: 50–55% (data from Globocan 2012 [1]). There is an urgent need for novel and personalized therapies to improve this outcome. Recent advances in genomic analyses and drug screening techniques have allowed the discovery of new therapeutic targets in several cancers. This is true in the case of EGFR, whose amplification and/or overexpression are frequent events in HNSCC. Although Cetuximab has been approved as an EGFR-targeted medicine for HNSCC treatment, few patients have shown long-term improvement with this treatment. Thus, alternative therapeutic strategies are under investigation. In the present study, we attempted to better define the mechanism of action of Bosutinib in head and neck cancer.

The analysis of large-scale sensitivity studies of pan-cancer cell lines to drugs under clinical and preclinical investigation revealed that Bosutinib is a candidate drug to treat HNSCC [11,12,13]. These studies suggested that EGFR could be a molecular marker of sensitivity to Bosutinib. It has also been reported that some anti-Src agents, including Bosutinib, could exert a direct effect on EGFR in other cell types [16]. Our findings showed that Bosutinib can inhibit cell growth in a panel of HNSCC cell lines. Bosutinib induced cell death by apoptosis, and the effect was more pronounced in the most sensitive cell lines, indicating that the intrinsic properties and alterations of the cells could affect the mechanism of action of the drug. Treatment with Bosutinib revealed different levels of sensitivity among HNSCC cell lines which correlated with the level of the phosphorylated-active form of EGFR. The most sensitive cell lines were those with the highest levels of active EGFR. This could be of help when selecting the group of patients that could benefit most from Bosutinib treatment. 

We showed that Bosutinib was very efficient in inhibiting EGFR and Src phosphorylation in HNSCC-sensitive cell lines. Similarly, Bosutinib achieved a complete blockade of EGFR and Src phosphorylation in HNSCC xenografts. Bosutinib decreased tumor growth, and the analysis of the tumors revealed that it reduced proliferation and activated apoptosis of the epithelial cells. In sensitive cell lines, Bosutinib inhibited ERK activation, but the Akt/mTOR pathway remained active. The combination of Bosutinib with the PI3Kα inhibitor Alpelisib resulted in the inactivation of both pathways as well as further reduction in cell viability as compared to either drug alone. Cells with alterations (mutation/amplification) in *PIK3CA* show increased sensitivity to Alpelisib [17]. We observed that the combination of both drugs was most effective in cells with mutations in the *PIK3CA* gene. Since treatment of *PIK3CA* mutant and wild type cells with Bosutinib and Alpelisib efficiently inhibited Akt and S6 activation, the differences in their sensitivity levels could be due to a distinct dependence of the cells on the PIK3/Akt/mTOR pathway for their survival.

In summary, the findings of the current work highlight the potential use of Bosutinib as a new drug for the treatment of HNSCC patients, especially for those with activation of the EGFR pathway. In addition, the concomitant administration of Bosutinib and PI3K inhibitors, such as Alpelisib, could be of potential benefit to patients with mutations in PI3Kα, thus providing a genotype-based treatment. This study provides a first step for future preclinical studies combining Bosutinib and Alpelisib in HNSCC.

## 4. Materials and Methods

### 4.1. Cell Lines and Inhibitors

A panel of six human HNSCC-derived cell lines was selected for the study (Table 1). Cal33, Detroit562, WSU-HN6, WSU-HN13 and WSU-HN17 were kindly provided by J. Silvio Gutkind (Department of Pharmacology and Moores Cancer Center, University of California, San Diego, La Jolla, CA, USA). The mutations in *EGFR* and *PIK3CA* were as described in Martin et al. [28] or based on data from Gutkind’s lab (WSU-HN17). RPMI2650 was purchased from the American Type Culture Collection (ATCC, Rockville, MD, USA). Cal33, WSU-HN6, WSU-HN13 and WSU-HN17 were cultured in DMEM (Gibco, Thermo Fisher Scientific, Waltham, MA, USA) supplemented with 10% fetal bovine serum (FBS, Gibco). Detroit562 and RPMI2650 were cultured in EMEM (Lonza, Basel, Switzerland) supplemented with 10% FBS. All cells were maintained at 37 °C in an atmosphere of 5% CO_2_ and 95% humidity.

For the in vitro studies, stock solutions of the inhibitors were prepared in DMSO following the manufacturer’s guidelines (Selleckchem, Houston, TX, USA) and taking into account the solubility of each compound. The stock concentrations used were 10 mg/mL (18.8 mM) for the Src/Abl tyrosine kinase inhibitor Bosutinib; 10 mg/mL (22.6 mM) for the PI3K inhibitor Alpelisib; 1 mg/mL (2.13 mM) for the PI3K inhibitor Dactolisib and 2.15 mg/mL (5 mM) for the EGFR inhibitor Erlotinib.

### 4.2. Cell Viability Assays and Drug Combination Studies

Cells were cultured in 96-well plates and, after 24 h of culturing, they were treated with escalating concentrations of Bosutinib or Alpelisib for 24 h. Cell viability was evaluated with the Colorimetric Assay XTT Cell Proliferation Kit II (Roche, Basel, Switzerland). Background absorbance (medium only) was subtracted, and the data (average of six replicates of each drug concentration) were normalized as percentages of vehicle control. Each experiment was performed at least three times, and each concentration point was replicated six times within each experiment. The concentrations of Bosutinib corresponding to its 25, 50 and 75 inhibitory concentrations (IC) (IC_25_, IC_50_ and IC_75_) were calculated with Graph Pad Prism5 software and are shown in Table 2. These values are defined as the concentration of drug causing decreases of 25%, 50% and 75% in cell viability, as measured by XTT, respectively, and they were used for the rest of the experiments in this manuscript (two-drug combination studies, cell cycle analysis and Western blotting). 

For the combined viability assays, we followed the method described by Chou and Talalay for two-drug combination studies [29] that calculates a “Combination Index” (CI) to quantitatively depict synergism (CI < 1), additive effects (CI = 1) and antagonism (CI > 1). Briefly, two-fold serial dilutions of the IC_50_ were performed for each drug alone and their mixture to create 4–5 concentrations. The combination of the two drugs was performed at a constant ratio. For this purpose, the IC_50_ concentration for Alpelisib for some of the HNSCC cell lines had to be established (Table 2). The CI was calculated using CompuSyn software (Available online: http://www.combosyn.com/).

### 4.3. Cell Cycle Analysis

The analysis of the cell cycle was performed by flow cytometry. For this purpose, cells were cultured in 12-well plates, and after 24 h, they were treated with their IC_25_, IC_50_ and IC_75._ After 24 h of treatment, cells were harvested, washed with PBS and fixed in 70% ice cold ethanol overnight at 4 °C. The samples were then centrifuged and suspended in PBS containing 2 µg/mL DAPI and 0.05% NP40 for at least 2 h. Finally, they were analyzed by a Becton Dickinson LSR Fortessa cell analyzer using BD FACSDiva software. Cell debris was excluded from the analysis based on the lower forward scatter/side scatter ratio (FSC-A/SCC-A ratio). Similarly, cell aggregates were excluded based on the FSC/FSC pulse width (FSC-A/FSC-W) and the Violet/Violet pulse width (405Violet450_50-A/405Violet450_50-W) gating. A minimum of 10,000 events were analyzed per sample. Data analyses of the G_0-1_, S, G_2_-M and the SubG_1_ (apoptosis) peaks were performed with FlowJo 7.6.5 software using the cell cycle analysis tool. Each experiment was performed at least twice, with 3 replicates of each concentration per experiment.

### 4.4. Copy Number Variation (CNV) Analysis

Genomic DNA was purified using the DNeasy Blood and Tissue Kit (Qiagen, Hilden, Germany) and quantified with a NanoDrop NP 1000 spectrophotometer (Thermo Scientific Nanodrop, Thermo Fisher, Waltham, MA, USA). Gene copy numbers were determined using 10 ng of DNA and the Taq-Man^®^ Copy Number Assay (Applied Biosystems, Foster City, CA, USA), following the manufacturer’s instructions. The probes for the target genes were labeled with FAM dye, and the probes for the control region were labeled with VIC dye. The qPCR reaction was run in a 7500 Fast Real-Time PCR System (Applied Biosystems) using the following amplification parameters: 2 min at 50 °C and 10 min at 95 °C, followed by 40 cycles of 15 s at 95 °C and 1 min at 60 °C. The copy number was calculated using the following equation: copy number = 2(ΔΔ*C*t), where ΔΔ*C*t = [Δ*C*t (unknown samples) − Δ*CC*t (known control)] and Δ*C*t = [*C*t (FAM) − *C*t (VIC)].

### 4.5. mRNA Expression Analysis

Total RNA was isolated using the miRNeasy Mini Kit (Qiagen) according to the manufacturer’s instructions, and genomic DNA was eliminated from the samples by DNase treatment (Rnase-Free Dnase Set, Qiagen). The amount of RNA was quantified as described for genomic DNA. Reverse transcription was performed using the Omniscript RT Kit (Qiagen). qPCR was performed in a 7500 Fast Real-Time PCR System (with the same amplification parameters as described for the CNV assays) using the Power SYBR GREEN PCR Master Mix (Applied Biosystems) and 1 µL of cDNA (50 ng) as the template. The reaction efficiency was calculated for each primer combination, and the β-glucuronidase (*GUSB*) housekeeping gene was used as the internal reference gene for normalization. The sequences of primers used were as follows: for *PIK3CA*, forward “GGCTCAAAGACAAGAACAAAGG” and reverse “TCCAGCACATGAACGTGTAAA”; for *EGFR*, forward “CATGTCGATGGACTTCCAGA” and reverse “GGGACAGCTTGGATCACACT”, for *GUSB*, forward “CGCCCTGCCTATCTGTATTC” and reverse “TCCCCACAGGGAGTGTGTAG”.

### 4.6. Western Blotting

Protein extracts were obtained using a lysis buffer (Hepes 40 mM, Triton-100 2%, β-glycerophosphate 80 mM, NaCl 200 mM, MgCl_2_ 40 mM, EGTA 20 mM) supplemented with protease and phosphatase inhibitor cocktails (Roche). Proteins were separated in 4–12% NuPAGE polyacrylamide gels (Invitrogen, Carlsbad, CA, USA) and then transferred to nitrocellulose membranes (GE Healthcare, Little Chalfont, UK) under wet conditions. Membranes were blocked in 5% non-fat milk in TBS-Tween (Tris-HCl 20 mM, NaCl 137 mM, 0.5% Tween) and then incubated overnight at 4 °C with the corresponding primary antibodies in 2.5% BSA TBS-Tween. Peroxidase-coupled secondary antibodies were used, specific for rabbit IgG (1/5000, GE Healthcare), goat IgG (1/10,000; Santa Cruz Biotechnology, Dallas, TX, USA) and mouse IgG (1/5000; Jackson, West Grove, PA, USA). The protein bands were detected using Super Signal Western Picoluminiscence Substrate (Pierce, Thermo Fisher) according to the manufacturer’s instructions, and they were quantified with Image Lab 5.2.2 software (BioRad, Hercules, CA, USA). To avoid cross-signal contamination in the analysis of protein phosphorylation, equal amounts of each protein lysate were loaded on two different gels and run in parallel. One of the gels was blotted with antibodies against the phosphorylated residues of the protein and the other with antibodies recognizing the total form of the protein. Primary antibodies against the following proteins of phosphoproteins were used: p-Src Tyr416 (clone D49G4), Src (clone 32G6), p-EGFR Tyr1068 (clone D7A5), p-EGFR Tyr845 (clone D63B4), EGFR (clone D38B1), p-Akt Ser473 (clone DE9), p-Akt Thr308 (clone 244F9), p-S6 Ser235/236 (clone D68F8), p-S6 Ser240/244 (clone D68F8) and S6 (clone 54D2) from Cell signaling; and Akt (clone N-19), ERK (clone K-23), p-ERK1/2 Thr202/Tyr204 (clone E-4) and β-actin (clone I-19) from Santa Cruz Biotechnology.

### 4.7. HNSCC Tumor-Derived Cell Line Xenograft Mouse Model

Cal33 cells were trypsinized and suspended in a mixure (2:1) of PBS with Matrigel (BD Biosciences, San Jose, CA, USA). Five million Cal33 cells in a total volume of 150 μL of PBS–Matrigel suspension were subcutaneously injected in each flank of (*n* = 18) 5–6-week-old immunocompromised nude (nu/nu) mice (Janvier, Saint-Berthevin, France). After 1 week of injection, tumors were visible. A more detailed description of the Cal33 tumors can be found in Supplementary Figure S2. Tumor growth was followed by measurements twice a week with a digital caliper, and tumor volume was calculated using the formula 0.5 × length × width^2^ [30]. At a tumor volume of 200–250 mm^3^, mice were randomized in 3 groups (*n* = 6) to receive the different treatments as follows: vehicle (0.5% methylcellulose 0.4% Tween 80), Bosutinib (Selleckchem) and Cetuximab (Merk Serono, Darmstadt, Germany). Bosutinib was dissolved in the vehicle, and 150 mg/kg was administrated via oral gavage (*per os*, p.o.) daily. Cetuximab was injected intraperitoneally (i.p.) every 5 days at a dose of 20 mg/kg. The Bosutinib and Cetuximab concentrations were chosen based on published data [17,31].

After 10 days of treatment, mice were sacrificed, and tumors were collected and preserved in 4% PBS-buffered formalin or 70% ethanol for further immunohistochemistry and immunofluorescence analysis, or in liquid nitrogen for Western blot analysis. All animal experiments were conducted in compliance with CIEMAT guidelines, and approved by the Animal Welfare Department of the Comunidad de Madrid (project reference: PROEX 183/15, approved 19/06/2015).

### 4.8. Immunohistochemistry and Immunofluorescence

Samples fixed in formalin or 70% ethanol were embedded in paraffin wax and sectioned (5 μm). Sections were stained with hematoxylin and eosin (H & E) or processed for immunohistochemistry and immunofluorescence using standard protocols. Mice underwent intraperitoneal injection (i.p.) with bromodeoxyuridine (BrdU; 0.1 mg/g weight in 0.9% NaCl; Roche) 1 h before sacrifice. Immunohistochemistry and immunofluorescence were done with primary antibodies against BrdU (Roche), pan-cytokeratin AE1/AE3 (Abcam, Cambridge, UK), cytokeratin 5 (K5) (Covance, Princeton, NJ, USA) and cleaved caspase-3 (Cell Signaling, Danvers, Massachusetts, USA). Secondary peroxidase complexed antibodies (Jackson, Cambridge, UK) were used in the immunohistochemistry studies, while fluorochrome-complexed secondary antibodies (Molecular Probes, Eugene, OR, USA) were used for immunofluorescence. Immunohistochemistries were counterstained with hematoxylin, and immunofluorescences were counterstained with DAPI (Roche).

### 4.9. Statistical Analysis

The IC_50_ of each cell line was estimated graphically as the concentration of the drug causing 50% loss in cell viability in XTT assays, using a non-linear regression curve fit in GraphPadPrism5 software. IC_25_ and IC_75_ values were also extrapolated from these curves. Data are shown as means ± standard errors of the mean (SEM). A Pearson correlation analysis was performed using GraphPadPrism, and *p* < 0.05 was considered statistically significant. In order to evaluate the effect of the dual treatment with Bosutinib and Alpelisib, drug combination experiments were designed as described by Chou and Talalay [29] and resultant data were analyzed with CompuSyn software [32]. A synergistic effect was considered when the Combination Index (CI) was below 0.7.

## Figures and Tables

**Table 1:** HNSCC cell lines.

Cell Line	Origin	*EGFR*	*PIK3CA*
Cal33	Tongue SCC	Wt	Mut (H1047R)
Detroit562	Pharynx/pleural effusion metastasis	Wt	Mut (H1047R)
RPMI2650	Nasal septum metastasis	Wt	Wt
WSU-HN6	Base of tongue SCC	Wt	Wt
WSU-HN13	Tongue SCC	Wt	Wt
WSU-HN17	Lymph node	Wt	Mut (E542K)

WSU: Wayne State University; SCC: squamous cell carcinoma; Mut/Wt: with/without (wild type) mutation in the specified gene

**Table 2:** Inhibitory concentration (IC) values for Bosutinib and Alpelisib (µM), combination index for both drugs at their IC_50_ and percentage of cells with SubG_1_ DNA content when treated with the IC_75_ of Bosutinib.

Cell Line	Bosutinib			Alpelisib IC_50_	Combination Index IC_50_	% of SubG1 Cells at IC_75_ of Bosutinib
	IC_25_	IC_50_	IC_75_			
WSU-HN17 *	14.42 ± 3.43 ^†^	18.56 ± 2.49 ^‡^	25.02 ± 2.09 ^‡^	-	-	44.25 ± 3.09 ^‡^
RPMI2650	10.89 ± 5.50	14.81 ± 4.31 ^‡^	23.82 ± 3.03 ^‡^	-	-	35.53 ± 0.63 ^‡^
Detroit562 *	8.27 ± 0.92	13.25 ± 0.52 ^‡^	21.52 ± 1.27 ^‡^	36.84 ± 6.79	0.25	26.76 ± 1.05 ^‡^
WSU-HN13	5.07 ± 1.12	6.49 ± 0.68	8.57 ± 0.18	35.41 ± 6.84	0.73	59.14 ± 2.11 ^†^
Cal33 *	2.98 ± 0.10	6.09 ± 0.11	12.76 ± 0.09	12.67 ± 2.39	0.63	77.17 ±1.31 ^‡^
WSU-HN6	2.94 ± 0.16	5.78 ± 0.29	11.35 ± 0.55	35.60 ± 4.60	0.74	67.17 ± 1.51

* Cell lines with mutations in *PIK3CA.* In red are Bosutinib-resistant cell lines. In blue are Bosutinib-sensitive cell lines. IC values are defined as the concentration of drug causing a decrease of 25% (IC_25_), 50% (IC_50_) and 75% (IC_75_) of cell viability as measured by XTT, respectively. Data are means ± SEMs from at least three different experiments for each cell line (each concentration point was replicated six times within each experiment). ^†^ *p* < 0.05, ^‡^ *p* < 0.01 compared to the respective value in WSU-HN6 (one-way ANOVA with Bonferroni post-hoc test).

## Supplementary Materials

Supplementary materials can be found at http://www.mdpi.com/1422-0067/19/7/1824/s1.

## Founding

This work was supported by FEDER cofounded MINECO [grant number SAF2015-66015-R] and ISCIII [grant numbers CB16/12/00228, PIE15/00076].

## Abbreviations

EGFR	Epidermal growth factor receptor
HNSCC	Head and neck squamous cell carcinoma
PIK3CA	Phosphatidylinositol-4,5-bisphosphate 3-kinase catalytic subunit α
PI3Kα	Phosphatidylinositol 3-kinase α
P.o.	*Per Os* (oral administration)
I.p.	Intraperitoneal
