# Supplementary material for: Bosutinib Inhibits EGFR Activation in Head and Neck Cancer
Source: Int J Mol Sci. 2018 Jun 21;19(7):1824. doi: 10.3390/ijms19071824 (PMC6073167; doi:10.3390/ijms19071824)
Supplement: Supplementary file 1 [file ijms-19-01824-s001.zip › Supplementary Figures IJMS_CLorz.pptx]

## Slide 1
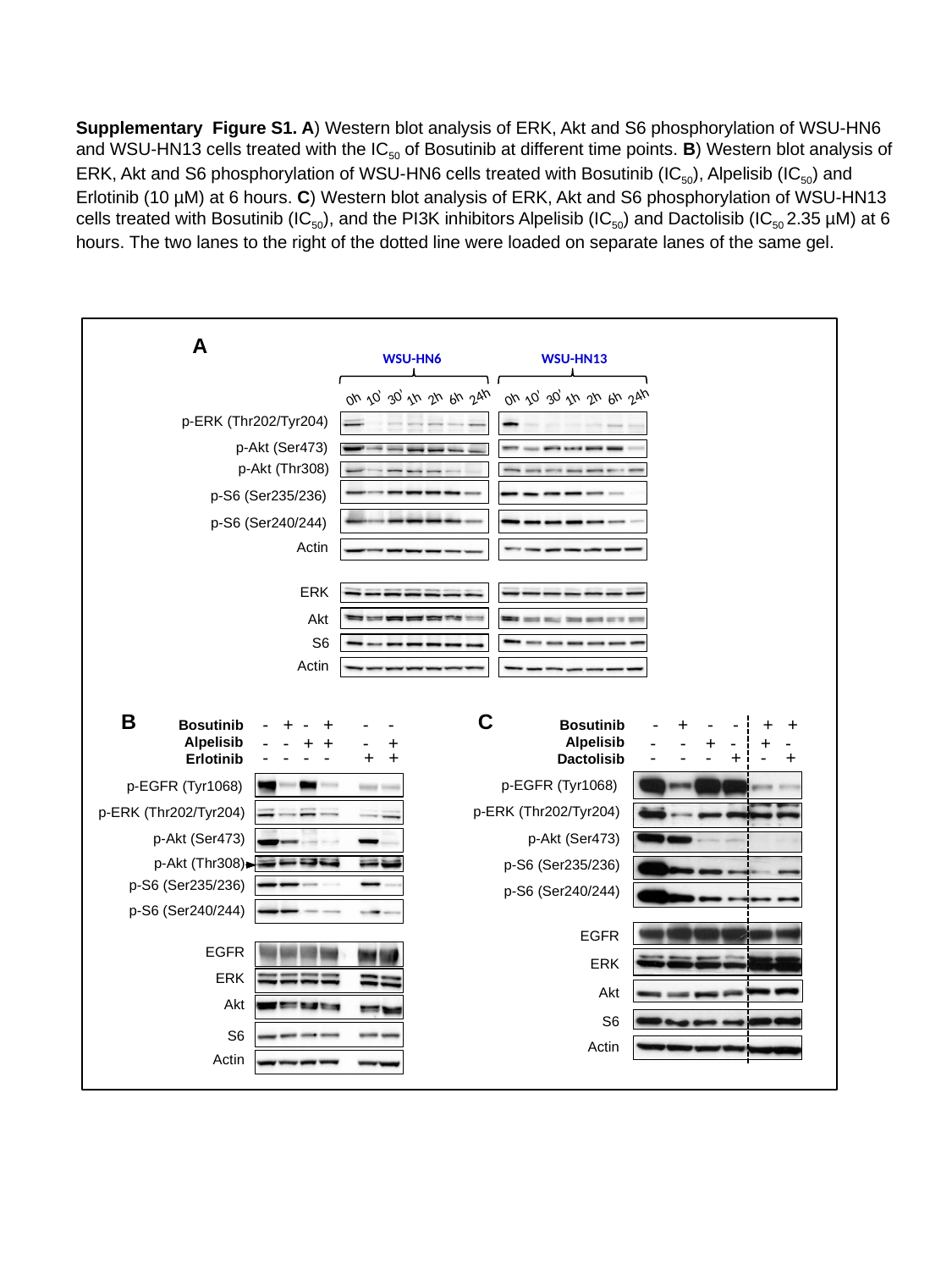

Supplementary Figure S1. A) Western blot analysis of ERK, Akt and S6 phosphorylation of WSU-HN6 and WSU-HN13 cells treated with the IC50 of Bosutinib at different time points. B) Western blot analysis of ERK, Akt and S6 phosphorylation of WSU-HN6 cells treated with Bosutinib (IC50), Alpelisib (IC50) and Erlotinib (10 µM) at 6 hours. C) Western blot analysis of ERK, Akt and S6 phosphorylation of WSU-HN13 cells treated with Bosutinib (IC50), and the PI3K inhibitors Alpelisib (IC50) and Dactolisib (IC50 2.35 µM) at 6 hours. The two lanes to the right of the dotted line were loaded on separate lanes of the same gel.
A
WSU-HN6
WSU-HN13
30’
24h
6h
2h
1h
0h
10’
30’
24h
6h
2h
1h
0h
10’
p-ERK (Thr202/Tyr204)
p-Akt (Ser473)
p-Akt (Thr308)
p-S6 (Ser235/236)
p-S6 (Ser240/244)
Actin
ERK
Akt
S6
Actin
B
C
- + - + - -
Bosutinib
- - + + - +
Alpelisib
- - - - + +
Erlotinib
p-EGFR (Tyr1068)
p-ERK (Thr202/Tyr204)
p-Akt (Ser473)
p-Akt (Thr308)
p-S6 (Ser235/236)
p-S6 (Ser240/244)
EGFR
ERK
Akt
S6
Actin
- + - - + +
Bosutinib
- - + - + -
Alpelisib
- - - + - +
Dactolisib
p-EGFR (Tyr1068)
p-ERK (Thr202/Tyr204)
p-Akt (Ser473)
p-S6 (Ser235/236)
p-S6 (Ser240/244)
EGFR
ERK
Akt
S6
Actin

## Slide 2
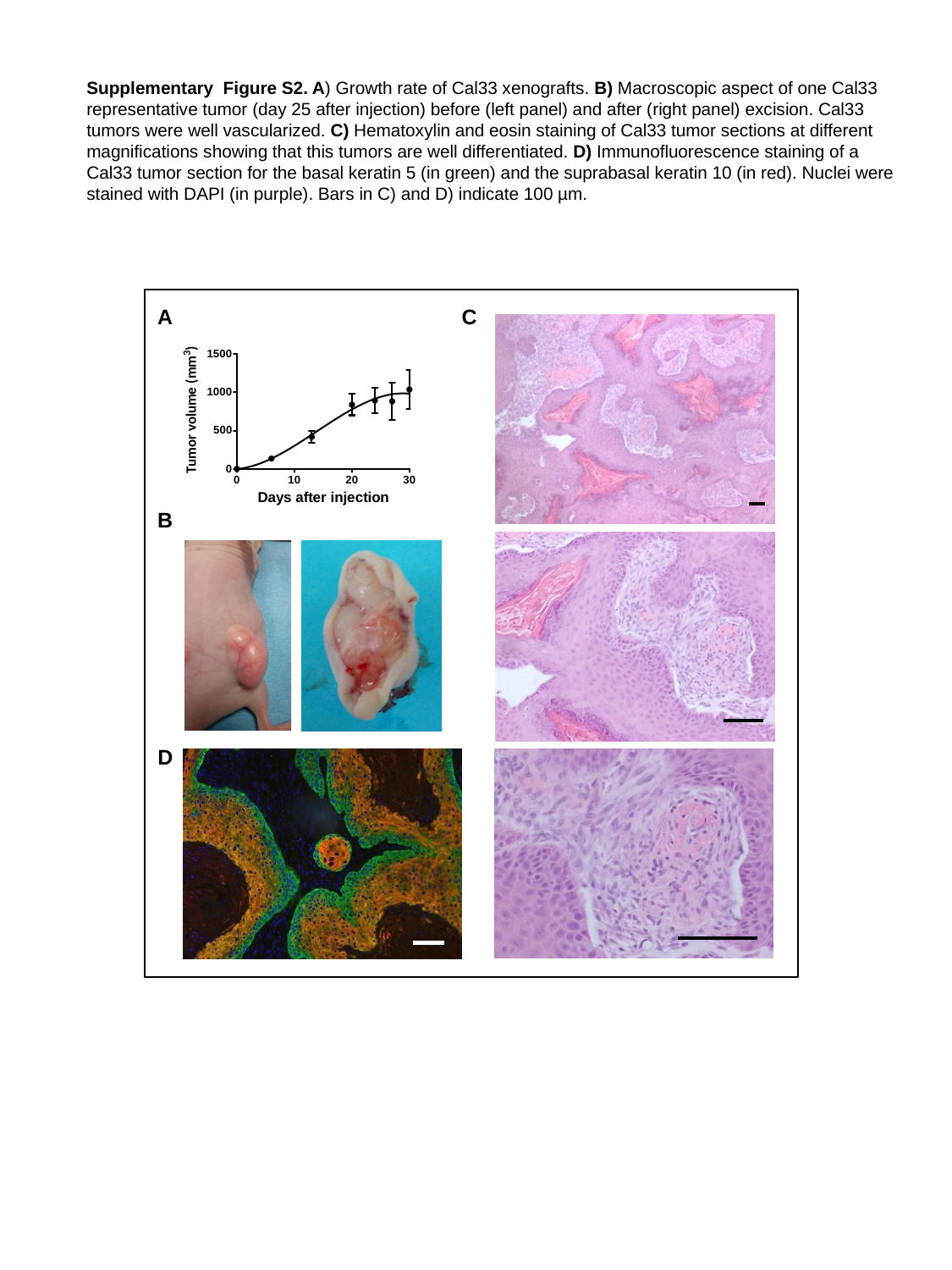

Supplementary Figure S2. A) Growth rate of Cal33 xenografts. B) Macroscopic aspect of one Cal33 representative tumor (day 25 after injection) before (left panel) and after (right panel) excision. Cal33 tumors were well vascularized. C) Hematoxylin and eosin staining of Cal33 tumor sections at different magnifications showing that this tumors are well differentiated. D) Immunofluorescence staining of a Cal33 tumor section for the basal keratin 5 (in green) and the suprabasal keratin 10 (in red). Nuclei were stained with DAPI (in purple). Bars in C) and D) indicate 100 µm.
A
C
B
D
